# Annotations

**Published:** 1904-05-21

**Authors:** 


### Annotations.

### International Home Relief Congress.

This Congress, which is to meet in Edinburgh in the early part of June, is an expression of a movement which must command the interest of all concerned in the advance of social reform. The object of the Congress is to compare the different ways of administering the various forms of public and private charity by those in charge of funds devoted to these purposes. The discussions will not include any reference to the administration of charitable relief in hospitals or other institutions in which those to be relieved are received in larger or smaller numbers. The immediate object of concern is rather the appropriate application of charitable measures for the preservation of the homes of those who from various causes are brought into a condition of want or distress. It is notorious that much well-intentioned charity in this direction is badly organised and directed, and that both private and public schemes are deficient in precision and definition. The Congress for promoting home relief hopes to supply these deficiencies and so to direct the available measures of assistance as to secure the best possible results from their application. There is no suggestion that large institutions such as model lodging-houses and the like have not an appropriate place in the scheme of charitable organisation, but it is felt that a better aim, when this is possible, is to retain the home and the influences which home life secures. There is every prospect of the forthcoming Congress attaining a decided success. Her Majesty, Queen Alexandra, has consented to be the Patron of the Congress, representatives are expected from the United States and the principal Continental countries, and the Local Government Board has authorised Parish Councils to send delegates at the charge of the public funds. The work of the Congress is to be conducted in five sections, namely: children, old age, able-bodied adults, sick adults, and insane and epileptics.

### Intestinal Parasites and their Symptoms.

The popular maternal practice of suspecting the presence of "worms" as the cause of many of the less serious disorders of childhood, and the fact that the event usually provides little or no justification for this opinion, are apt to excite in the professional mind an incredulous attitude towards the proposition that intestinal parasites may be responsible for many and varied symptoms. This attitude is further supported by the numerous cases in which, without any previous disturbance, the parasites or their ova are discovered in the stools. That in many, perhaps in the majority of cases, the presence of worms in the intestine is unattended by any appreciable prejudice to health may be accepted as certain. It is equally certain that every now and then very marked symptoms disappear after the expulsion of some form of intestinal parasite, and the conclusion is irresistible that such symptoms are caused by the parasites. The presumption is that the parasite may either cause disturbance in the nervous system by reflex irritation, or may produce some form of poison which becomes absorbed and depreciates the quality of the blood. In this latter respect it may be remembered that the suggestion has been made that pernicious anæmia may be caused by the presence of a tapeworm in the intestine. At occasional intervals cases are recorded which illustrate the above-mentioned clinical possibilities, and it is well that the practical lesson they enforce should be borne in mind. Within the last few weeks Dr. Jas. Burnett, of Edinburgh, has reported two cases of chorea, in each of which the symptoms ceased on expulsion of a tape-worm. Another record is that of a girl with considerable anæmia for which no explanation could be found until a tape-worm was passed, after which the patient rapidly recovered. In this last instance it is of interest to note that the examination of the blood showed considerable increase of the eosinophile cells. This condition with more or less leucocytosis has been emphasised in trichiniasis, but later observations tend to show that these blood changes may be noted in connection with various forms of intestinal parasites.

### Eugenics.

On Monday last, in the School of Economics, a meeting of the Sociological Society was held in order to discuss a paper read by Dr. Francis Galton, F.R.S., on "Eugenics; its definition, scope and aims." The author defined eugenics as the science which deals with all influences that improve the inborn qualities of the race; also with those that develop them to the utmost advantage. Such a definition is apt at once to conjure up the vision of rich pastures and harvest fields whose fruits are trampled into the ground by the feet of contending factions and ruined by controversial war. However, Dr. Galton thinks that the aims of eugenics may be modified so as to avoid the strife of conflicting ideals by piercing deeper than these and considering only those fundamental qualities which the great majority of thoughtful men will estimate as worthy of aspiration. For example: few will quarrel with the statement that it is better to be healthy than sick, vigorous than weak, well fitted than ill fitted for life's struggle, and it is to such problems as these that the science of eugenics is applied. Most people will appreciate the value of bestowing more attention to these questions, even if their enthusiasm falls short of Dr. Galton's, who thinks that eugenics has strong claims to become an orthodox religious tenet of the future. Doubtless a desire to improve the race is equally as necessary and certainly not more easy to create than a knowledge of how such an end may be achieved; and here it may fairly be asked whether frivolity and idleness, sensuality and luxuriousness, artificiality and sterility, spreading from the upper to the lower social strata, and sapping like a great fungus the country's vitality, are not merely symptoms of national decline, while the real causes lie deeper down. Possibly this is a matter which the Sociological Society will take steps to elucidate on some future occasion. Broadly speaking, we take it to be the aim of eugenics to place the good of the race above individual considerations, and anything which is accomplished towards attaining this ideal will be, we think, a great step forward in the history of the human race.

